# 40-year-old Male with a Headache and Altered Mental Status

**DOI:** 10.5811/cpcem.2020.10.49546

**Published:** 2020-10-20

**Authors:** Rebecca J. Rubenstein, Leen Alblaihed, Zachary D.W. Dezman, Laura J. Bontempo

**Affiliations:** * University of Maryland Medical Center, Department of Emergency Medicine, Baltimore, Maryland; † University of Maryland School of Medicine, Department of Emergency Medicine, Baltimore, Maryland

## Abstract

A 40-year-old man presents to the emergency department with headache, nausea and paresthesias, with subsequent fever and mental status change. Magnetic resonance imaging showed increased fluid-attenuation inversion recovery signal involving multiple areas of the brain, including the pons. This case takes the reader through the differential diagnosis of rhombencephalitis (inflammation of the hindbrain) with discussion of the unanticipated ultimate diagnosis and its treatment.

## CASE PRESENTATION (Dr. Rubenstein)

A 40-year-old male was brought by a friend to the emergency department (ED) for evaluation with a chief complaint of headache. The patient reported a headache that was diffuse, severe in intensity, throbbing in nature, and gradual in onset. The headache started when he lay down to sleep that evening. It was associated with nausea and numbness and tingling in his bilateral hands and feet. The headache was preceded by several hours of fatigue and generalized weakness. The patient drank six 12-ounce beers before going to bed. The friend at the bedside explained that he typically drank that amount, and expressed concern because the patient seemed much more intoxicated than he typically would be after drinking that amount.

The patient had a past medical history of depression and anxiety. He had a surgical history of an open reduction and internal fixation of a right calcaneal fracture 10 years prior. He took sertraline 100 milligrams (mg) daily, and had no drug allergies. He smoked one pack of cigarettes per day and was a daily drinker, but had not experienced alcohol withdrawal. He denied use of illicit drugs. Family history was notable for a cerebrovascular accident and myocardial infarction in his parents, and vertigo in a sibling. He worked as a contractor for a gas and electric company, and spent most of his day outside. He had no recent travel history. A review of systems revealed a one-week history of right ankle pain, swelling, and redness that the patient attributed to a “spider bite.”

The patient was alert, oriented, and uncomfortable but non-toxic appearing on the initial exam. His temperature was 36.8° Celsius (C), with a heart rate of 71 beats per minute, blood pressure (BP) of 139/83 millimeters (mm) of mercury, and an oxygen saturation of 98% on room air with a respiratory rate of 16 breaths per minute. He weighed 82.6 kilograms (kg), was 1.96 meters tall with a body mass index of 22.5 kg/m^2^. He appeared well developed and well nourished. His head was normocephalic and atraumatic. He had moist mucous membranes, without oral lesions, and with a normal oropharynx. Pupils were 2 mm, equal, and sluggishly reactive to light. His extraocular movements were normal. There was no scleral icterus or conjunctival pallor. Visual acuity was grossly normal. The neck was supple without cervical lymphadenopathy, meningismus, or midline or paraspinal cervical spine tenderness. Heart was regular rate and rhythm, without murmur, rubs, or gallops. Breath sounds were clear bilaterally without wheezing, rhonchi, or rales. Abdomen was soft and nontender, without rebound, guarding, or rigidity, and with normoactive bowel sounds. Extremities were warm and well perfused with good distal pulses.

There was a small area of blanching erythema and ecchymosis in the right lateral malleolar region. There was no increased warmth, edema, other evidence of trauma, or painful or limited range of motion of the ankle. Neurologic exam showed cranial nerves II–XII intact, 4/5 strength throughout the bilateral upper extremities, 5/5 strength throughout the bilateral lower extremities, with normal tone, and normal sensation throughout. Speech was clear and fluent, and mood and affect were appropriate. Skin was warm and dry. A papular rash was noted on his lower extremities (Image). It did not involve the palms or soles.

The patient’s initial laboratory testing results are shown in Table 1. His electrocardiogram showed a normal sinus rhythm, with normal axis, normal intervals, and no ST-segment elevation, depression, or T-wave inversions. Two hours after his initial evaluation, he began vomiting and complained of dysarthria, dysphagia, and vertigo. Tongue fasciculations were present. He received a dose of metoclopramide. Due to concern for an acute cerebral vascular accident, a computed tomography (CT) of the head, and a CT angiography of the head and neck were subsequently performed and showed no acute abnormality. Neurology was consulted, and a magnetic resonance imaging (MRI) of the brain without contrast was obtained per their recommendation. The MRI was markedly limited by motion artifact and was of poor diagnostic quality. As described by radiology, the image showed foci of increased fluid-attenuation inversion recovery (FLAIR) signal involving portions of the cortex of the frontal lobes bilaterally, deep periventricular white matter, left side of midbrain, throughout the pons, and in the deep left cerebellar hemisphere.

The patient was reassessed when he returned from MRI. His BP and heart rate were relatively unchanged, but he was febrile (38.4°C). The patient had become somnolent and he was no longer managing his oral secretions. It was determined that he was no longer adequately protecting his airway. He was intubated without complication, blood cultures were drawn, and he was started on empiric broad-spectrum intravenous (IV) antibiotics. A lumbar puncture was performed and cerebrospinal fluid (CSF) was obtained. The results are shown in Table 2. A diagnostic test was sent, which confirmed the diagnosis.

## CASE DISCUSSION (Dr. Alblaihed)

Headache is one of the most common complaints seen in the ED. When a patient presents with headache, fever, and neurological symptoms, such as altered mental status, this is extremely concerning for encephalitis or meningoencephalitis.

I began by looking at the causes of altered mental status. These can include anoxic or ischemic insults, metabolic derangements, nutritional deficiency, trauma, and toxins or medications effects. Systemic infections can present as delirium, while traumatic brain injury, malignant hypertension, and seizures can cause encephalopathy. Rare causes include Hashimoto’s encephalopathy, mitochondrial cytopathy, and paraneoplastic syndromes.^1^ I went back to see if there were clues in the patient presentation that could help me narrow down this wide differential diagnosis.

The patient presented with one week of ankle pain, redness, and swelling due to a “possible bite.” He had suddenly developed fatigue, generalized weakness, and muscle aches over the prior day. He then began to complain of a headache that was severe, generalized, throbbing, and started when he lay down. This headache was associated with nausea and paresthesias. On exam he had sluggish pupils, although this is nonspecific. His ankle showed normal range of motion but also had a small area of ecchymosis and erythema (possible bite). He had a rash. His neurological exam was incomplete. It would have been helpful to know details of the cerebellar exam since the patient had complained of vertigo. It is unclear whether the patient had a normal gait or if nystagmus was present. Regarding the vital signs and laboratory results, nothing was overtly concerning.

During his ED evaluation he became dizzy, dysarthric, and had dysphagia. He was treated with IV fluids (IVF) and metoclopramide. This raised my concern for serotonin syndrome, especially because he was taking sertraline at home. It was reassuring that there were no reports of muscle stiffness or rigidity, hyperthermia, tachycardia, dilated pupils, or hypertension. Another concern was that the patient developed central pontine myelinolysis (CPM) from the IVF. Although he did not suffer from hyponatremia, CPM can occur unrelated to the sodium concentration in malnourished, chronic alcohol abusers by means of increasing the extracellular osmotic pressure upon refeeding. However, this patient had no history of starvation or malnutrition. The CT and CT angiography that were done were reportedly normal.

Within hours of his presentation to the ED, he developed tongue fasciculation which, to me, usually means alcohol withdrawal; however, there are many other causes such as lower motor neuron disease, muscle-specific receptor tyrosine kinase, myasthenia gravis, brainstem lesions, base of skull tumors, radiation of the skull base, unilateral hypoglossal neuropathy, and syringomyelia.^2^ Following his MRI, he required intubation for altered mental status. His course was acute and rapidly progressing. This will help me eliminate chronic processes as a cause for his presentation.

Other things I noticed were that he was febrile, with a normal heart rate, oxygen saturation, and BP. Fever without tachycardia (Faget sign) is associated with several tick- and mosquito-borne illnesses. The patient had CSF results that were nonspecific, most likely viral etiology; however, with “clearing” of the red blood cells, it is unlikely to be herpes simplex virus. The MRI showed increased FLAIR signal to the pons (more on the left), frontal subcortical, genu of the corpus callosum, frontal parasagittal, and the periventricular as well as cerebellar (left) areas. These are findings of atypical rhombencephalitis (hindbrain).

### To summarize

This is a 40-year-old man with a fever, headache, neurological symptoms, rash, ankle swelling, and tongue fasciculations whose symptoms progress to bulbar weakness requiring intubation. His MRI is concerning for rhombencephalitis.

Putting the clinical picture together, the MRI was my biggest clue to narrowing down my differential diagnosis. Rhombencephalitis, inflammation affecting the hindbrain, was present on the MRI as well as frontal and periventricular FLAIR. Adding to that the acuity of presentation, and the possible “bite” to the ankle, I came up with a list of diagnoses (Table 3).

The causes of rhombencephalitis that are consistent with this patient’s presentation and, therefore, remain on my differential diagnosis are the following (similarities with the case are in **bold**):

- West Nile encephalitis (WNE):^4^
  - Endemic to the United States, typically through mosquito **bite**
  - **Rapid** onset
  - “Flu-like symptoms” with nausea, vomiting, myalgias, **fever**, **headache, rash, tongue fasciculations**
  - Neurological symptoms include lower motor neuron **weakness**, flaccid paralysis, and paresthesias
  - MRI findings show abnormalities in the **pons**
  - White blood cell count is usually **normal**
  - CSF is nonspecific but may show a **viral picture** similar to this patient’s
- Eastern equine encephalitis (EEE) (Acute disseminated encephalomyelitis):^5^
  - Extremely rare, typically transmitted through mosquito or snake **bite** (copperhead and cottonmouth)
  - 4–10 days incubation
  - **Fever**, chills, malaise, arthralgia, and myalgia
  - Encephalitis develops after several days
  - Diagnosed by detecting EEE virus immunoglobulin (Ig) M in CSF
- Listeria rhombencephalitis:^6^
  - The most common cause of rhombencephalitis, usually in immunocompetent patients
  - Biphasic illness.
    - Prodrome of <16 days, **fever**, nausea, vomiting, **headache**
    - Followed by neurological symptoms: cranial nerve palsy (VI, VII), cerebellar dysfunction, **motor dysfunction**, sensory dysfunction, **altered mental status**
  - Rash can be present
  - CSF analysis shows:
    - Neutrophilia and lymphocytosis
    - Increased protein with normal glucose
    - Cultures can be negative
  - Polymerase chain reaction (PCR) may be negative
  - MRI is diagnostic: predilection for the dorsal **brain stem** and cerebellum, specifically the floor of the fourth ventricle.
- Rocky Mountain spotted fever (RMSF) encephalitis:
  - Transmitted via tick **bite**
  - Progresses over days
  - **Fever**, **headache**, myalgia, nausea
  - **Delayed petechial rash starting over the ankles** and wrists
  - **Joint swelling** can be present
  - Neurological symptoms including ataxia, seizures, dysarthria
  - MRI: increased intensity in perivascular spaces^7^
  - Labs will show thrombocytopenia, elevated transaminases, hyponatremia
- Lyme neuroborreliosis:
  - Encephalitis is rare in Lyme disease
  - Slower onset
  - **Fever**, myalgia, **headache**, arthritis, **rash**
  - Diagnosed with Lyme PCR in CSF; it has variable sensitivity
  - MRI: foci of **periventricular** / **subcortica**l T2 hyperintensity, nerve root enhancement, and meningeal enhancement^8^
- Anaplasmosis (human granulocytic ehrlichiosis):^9^
  - Symptoms range from asymptomatic to fatal
  - Tick bite ↔ 5-day incubation ↔ fever, myalgia, **headache**, nausea, arthralgia, possibly **rash**
  - Neurological symptoms include facial palsy, demyelinating polyneuropathy, brachial plexopathy
  - Rarely involves the central nervous system
  - Leukopenia, thrombocytopenia, elevated liver enzymes
  - Wright or Giemsa-stained blood smears (25–75% sensitive)
  - PCR up to 90% sensitive before antibiotics are given
  - Diagnosed by detecting IgM, IgG by immunofluorescence
- Ehrlichiosis:
  - Symptoms start 1–2 weeks after tick bite
  - **Commonly fever**, chills, **severe headache**, myalgias, and a maculopapular rash
  - Less common symptoms include **nausea**, vomiting, and confusion
  - Can include meningoencephalitis, seizures and coma
  - Rarely peripheral neuropathies and cranial neuritis
- *Borrelia miyamotoi*:
  - Transmitted via the same tick as Lyme disease.
  - Similar symptoms as Lyme disease (minus rash)
  - **Fever**, **headache**, fatigue, myalgia
  - Labs may be normal, or may show leukopenia, thrombocytopenia, elevated liver enzymes, proteinuria
  - Detectable by PCR
- Q fever (*Coxiella burnetii*):
  - **Fever**, **headache**, sore throat, malaise, nausea, diarrhea, chest pain, nonproductive cough, pneumonia, and hepatitis
  - Neurological manifestations occur in about 1% of patients and include meningitis, **encephalitis**, myelitis and/or peripheral neuropathy
  - Detected by indirect immunofluorescence assay.

Putting together the patient’s clinical presentation, I think the most likely cause is an infectious encephalitis due to a tick bite. As with many other tick-borne diseases, the symptoms and labs are largely non-specific, thus confounding the diagnosis and making it difficult to pinpoint the exact etiology. Based on the reasoning above, the items remaining on my differential diagnosis are RMSF, WNE, listeria rhombencephalitis, anaplasmosis, Q fever, ehrlichiosis, and Lyme neuroborreliosis. Although neurological involvement in West Nile virus (WNV) encephalitis is <1%, patients who have the disease have tongue fasciculations, lower motor neuron disease and weakness, and local paresthesia. It has a rapid progression, similar to this patient, and the MRI findings involve the pons, which is also similar to this patient. I conclude that **WNV is the culprit in this case**, and the **diagnostic test would be a CSF IgM for WNV**.

## CASE OUTCOME (Dr. Rubenstein)

The diagnostic test was an Ehrlichia PCR, which detected *Ehrlichia ewingii*. The patient was admitted to the intensive care unit (ICU), and was continued on vancomycin, ceftriaxone, ampicillin, and acyclovir. Per neurology and radiology, possible etiologies of the MRI findings included multifocal infectious processes, encephalitis, and acute disseminated encephalomyelitis.

While in the ICU, additional history was obtained from the patient’s wife. She reported that he had intermittent fevers for several days preceding admission and multiple recent tick and mosquito bites. She also stated that he had a several-year history of recurrent sinusitis, bronchitis, and pneumonia. He was subsequently started on doxycycline with concern for tick-borne illness. With a history of recurrent upper and lower respiratory tract infections, there was concern that an undiagnosed underlying immunodeficiency could have contributed to his severe course.

Unfortunately, over the next several hours, the patient’s clinical condition worsened. He was persistently hyperpyrexic despite antipyretics, BPs were labile, and neurologic exam revealed bilaterally fixed and dilated pupils. A repeat CT of the head was performed and showed interval development of significant edema, and a small area of hemorrhage with mass effect and midline shift. All sedating medications were held, and he remained unresponsive with loss of oculocephalic, corneal, and gag reflexes. An electroencephalogram was obtained and showed no variability or reactivity, further indicative of a poor prognosis.

After discussion with the family, and in consideration of the patient’s wishes, he was transitioned to comfort care with a plan for compassionate ventilator weaning. Within three days of his initial presentation, the patient died. The cause of death was hemorrhagic encephalitis leading to cerebral edema and brain herniation. Two days posthumously, the ehrlichia PCR resulted.

## RESIDENT DISCUSSION

Ehrlichiosis, a zoonosis, describes an illness caused by bacteria of the genus *Ehrlichia*, most commonly *E. chaffeensis*, and *E. ewingii*. They are obligate intracellular pathogens and were discovered to cause disease in humans in 1986, and in 1999, respectively.^10^,^11^ The incidence and prevalence of ehrlichiosis has been steadily increasing since it was discovered. The disease primarily occurs in the geographic distribution of its arthropod vector, the lone star tick (*Amblyomma americanum*): South Central, Midwest, and Eastern United States. The vertebrate reservoir for *E. chaffeensis* is deer, while *E. ewingii* is found in both deer and dogs. The peak transmission of Ehrlichia occurs in summer and peaks in June and July.^12^ Ehrlichiosis is overwhelmingly transmitted through tick bites, but the disease has been transmitted through blood transfusion, kidney transplantation, and direct contact with a slaughtered deer.^13^

The signs and symptoms of ehrlichiosis typically occur within 5–14 days after the bite of an infected tick. Signs and symptoms in the first few days of illness most commonly include fever, as well as headache, malaise, myalgias, and confusion. Rash occurs in less than 30% of adults and up to 60% of children. The rash is nonpruritic and can vary in appearance from petechial to maculopapular to macular. Late signs and symptoms include encephalitis, meningitis, coagulopathies, organ failure, and death. Risk factors for severe disease include an immunocompromised state, the extremes of age, and delayed treatment.^14^

The workup in the ED will depend on the presenting symptoms and severity of illness. It is important to note that the tick bite is not painful, and it is unlikely that your patient will remember being bitten. It is the job of the clinician to keep tick-borne illnesses such as ehrlichiosis on the differential diagnosis for patients presenting with nonspecific febrile illnesses in an endemic area, especially during peak transmission months in the summer. A thorough history should include a history of recent tick bites and exposure to wooded areas or high grass.

In a patient with fever, altered mental status, or headache of uncertain origin, a lumbar puncture should be considered. Most commonly, CSF analysis shows lymphocytic pleocytosis with elevated protein. The most common lab abnormalities found in patients with ehrlichiosis include leukopenia, thrombocytopenia, transaminitis, elevated lactate dehydrogenase, and elevated alkaline phosphatase. The CSF and laboratory findings are nonspecific and cannot definitively diagnose ehrlichiosis.

Treatment for ehrlichiosis should not be delayed by the lack of a diagnosis. If there is a suspicion, treatment should be started immediately. Ehrlichiosis can be diagnosed by PCR, serology, immunohistochemical assay and culture, and blood-smear microscopy; however, these are not universally available. Which test to send should be decided based on institutional availability and in consultation with an infectious disease expert.

Doxycycline is the treatment of choice for ehrlichiosis in patients of all ages. The dosing regimen for adults is 100 mg either orally or IV, twice daily. For children less than 45 kg, the dose is 2.2 mg/kg/dose, either IV or orally, twice daily. In a child less than or equal to eight years of age, doxycycline should still be administered, as the benefits of treatment outweigh the risks of potential adverse effects. There is evidence that a short course of doxycycline does not result in permanent teeth staining or enamel hypoplasia. The minimum recommended course of doxycycline is five to seven days, and treatment should continue at least three days after the subsidence of fever or until there is evidence of clinical improvement. In a critically ill patient, or in a patient with severe disease, typical broad-spectrum antibiotic coverage should be started along with doxycycline, as the presentation is nonspecific and can overlap with many other disease processes.

## FINAL DIAGNOSIS

Hemorrhagic encephalitis secondary to ehrlichiosis leading to cerebral edema and brain herniation.

## KEY TEACHING POINTS

- Ehrlichiosis must be considered for a patient with a febrile illness of unknown origin, especially in an endemic area during the summer months.
- Practitioners should maintain a high clinical suspicion for all tick-borne diseases in patients from endemic regions who present with nonspecific febrile illnesses.
- Endemic areas include the South Central, Midwest, and Eastern United States.
- Treatment of choice is doxycycline for patients of all ages.

## Figures and Tables

**Image:**
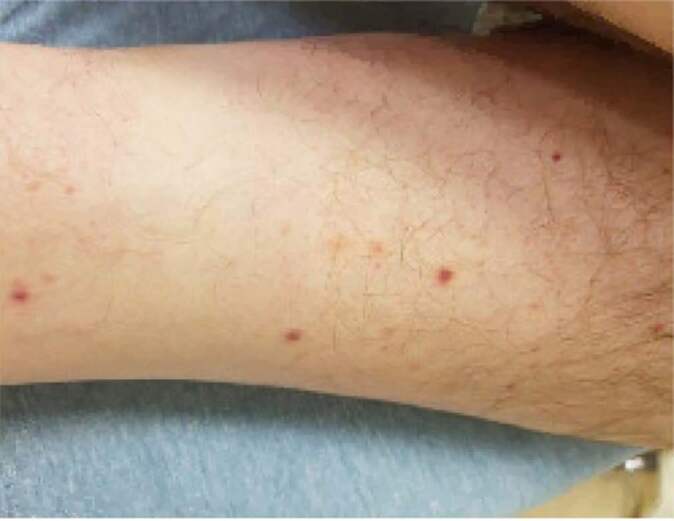
Left lower extremity rash of a 40-year-old male who presented with a headache and altered mental status.

**Table 1:** Blood laboratory results of a 40-year-old male with a headache and altered mental status.

Complete blood cell count	Patient value	Normal
White blood cells	9.4 K/mcL	4.8 – 10.9 K/mcl
Hemoglobin	13.4 g/dL	14 – 18 g/dL
Hematocrit	38.5 %	42.0 – 52.0 %
Platelets	244 K/mcL	130 – 400 K/mcL
Differential		
Polymorphonuclear leukocytes	46 %	
Lymphocytes	40 %	
Monocytes	13 %	
Eosinophils	0 %	
Serum chemistries		
Sodium	137 mmol/L	136 – 144 mmol/L
Potassium	3.4 mmol/L	3.5 – 5.3 mmol/L
Chloride	102 mmol/L	98 – 107 mmol/L
Bicarbonate	24 mmol/L	22 – 32 mmol/L
Anion gap	11	2 – 11
Blood urea nitrogen	11 mg/dL	7 – 25 mg/dL
Creatinine	0.77 mg/dL	0.90 – 1.30 mg/dL
Glucose	206 mg/dL	75 – 110 mg/dL
Calcium	8.9 mg/dL	8.6 – 10.3 mg/dL
Magnesium	1.7 mg/dL	1.8 – 2.5 mg/dL
Total protein	7.7 g/dL	6.0 – 8.1 g/dL
Albumin	3.9 g/dL	3.3 – 4.6 g/dL
Aspartate aminotransferase	26 IU/L	15 – 41 IU/L
Alanine aminotransferase	36 IU/L	7 – 52 IU/L
Alkaline phosphatase	72 IU/L	32 – 91 IU/L
Total bilirubin	36 mg/dL	0.10 – 1.30 mg/dL
Ammonia	36 umol/L	16 – 53 umol/L

*K*, thousand; *mcL*, microliter; *g*, grams; *dL*, deciliter; *mmol*, millimole; *L*, liter; mg, milligram; *IU*, international units; *umol*, micromole.

**Table 2:** Cerebrospinal fluid results of a 40-year-old male with a headache and altered mental status.

Test	Patient value	Normal
Glucose	102 mg/dL	50 – 80 mg/dL
Protein	177 mg/dL	15 – 45 mg/dL
White blood cells (WBC) (Tube 1, Tube 4)	39 mm3, 54 mm3	0 – 5 mm3
Red blood cells (Tube 1, Tube 4)	4 mm3, 0 mm3	0 – 5 mm3
Neutrophils (Tube 1, Tube 4)	96 %, 54 %	3 – 7 %
Lymphocytes (Tube 1, Tube 4)	2 %, 2 %	28 – 96 %
Monocytes (Tube 1, Tube 4)	2 %, 8 %	16 – 56 %
Gram stain	Few WBC. No organisms seen.	
Color	Colorless	
Clarity	Clear	

*dL*, deciliter; *mg*, milligram; *mm^3^*, cubic millimeters; *WBC*, white blood cells.

**Table 3:** Causes of rhombencephalitis.^3^

Infection	
Viral	Rabies Enterovirus 71 Herpes simplex virus (HSV) Epstein-Barr virus (EBV) Human herpesvirus 6 (HHV6) Flaviviruses (eg, West Nile virus and Japanese encephalitis virus) Eastern equine encephalitis
Bacterial	Listeria Mycobacterium tuberculosis Rickettsia, Borrelia burgdorferi, Salmonella typhi, Legionella bozemanii, and Mycoplasma pneumoniae (rarely causes encephalitis but can involve the brainstem) Pneumococcus
Autoimmune	Behçet disease (most common autoimmune cause) Multiple sclerosis Systemic lupus erythematosus Acute disseminated encephalomyelitis Progressive multifocal leukoencephalopathy Paraneoplastic syndromes
Other	Lymphoma (rare)
